# A retrospective analysis of the InterTan nail and proximal femoral nail anti-rotation in the treatment of intertrochanteric fractures in elderly patients with osteoporosis: a minimum follow-up of 3 years

**DOI:** 10.1186/s13018-017-0648-2

**Published:** 2017-10-10

**Authors:** Hui Zhang, Xiaoxiao Zhu, Genwang Pei, Xianshang Zeng, Nan Zhang, Ping Xu, Deng Chen, Weiguang Yu, Xinchao Zhang

**Affiliations:** 1 Emergency Department, The First Affiliated Hospital of Sun Yat-sen University, Huangpu East Road No. 183, Huangpu District, Guangzhou, Guangdong 510700 China; 2 Endocrine Department, The First Affiliated Hospital of Sun Yat-sen University, Huangpu East Road No. 183, Huangpu District, Guangzhou, Guangdong 510700 China; 3 Department of ENT, The First Affiliated Hospital of Sun Yat-sen University, Huangpu East Road No. 183, Huangpu District, Guangzhou, Guangdong 510700 China; 4 Department of Orthopedics, The First Affiliated Hospital of Sun Yat-sen University, Huangpu East Road No. 183, Huangpu District, Guangzhou, Guangdong 510700 China; 5 Department of Anesthesiology, The First Affiliated Hospital of Sun Yat-sen University, Huangpu East Road No. 183, Huangpu District, Guangzhou, Guangdong 510700 China; 6 Radiology Department, The First Affiliated Hospital of Sun Yat-sen University, Huangpu East Road No. 183, Huangpu District, Guangzhou, Guangdong 510700 China; 7 Department of Joint Surgery, The First People’s Hospital of Jingmen, Xiangshan Avenue No. 168, Dongbao District, Jingmen, Hubei 448000 China; 8 Department of Orthopaedics, Jinshan Hospital, Fudan University, Longhang Road No. 1508, Jinshan District, Shanghai City, 201508 China

**Keywords:** Intertrochanteric fracture, InterTAN nail, Proximal femoral nail anti-rotation, Harris Hip Score

## Abstract

**Background:**

The study aims to compare the long-term functional and radiographic outcomes of two devices for the treatment of primary intertrochanteric fractures (IFs, OTA 3.1A2.1–A2.3) in elderly patients with osteoporosis.

**Methods:**

Between December 2010 and August 2014, 332 elderly osteoporosis patients with IFs (OTA 3.1A2.1–A2.3) fixed by an InterTAN nail (IT) or a proximal femoral nail anti-rotation (PFNA) device were retrospectively evaluated. Follow-up occurred 1, 3, 6, and 12 months postoperatively and every year thereafter. Mortality was recorded. Patient-related functional and radiographic outcomes were compared. The primary endpoint was the long-term radiographic outcomes. The secondary endpoint was the long-term functional outcomes.

**Results:**

A total of 283 patients (283 hips) with osteoporosis (IT, *n* = 144; PFNA, *n* = 139) were evaluated with a mean follow-up period of 38.8 months (range, 36–43 months). No between-group significant differences were noted in the patient demographics, operation variables, and postoperative Harris Hip Score. More radiographic complications were noted in terms of screw cut-out, femoral shaft fracture distal or around the tip of the main nail, and varus collapse of the femoral head in the PFNA group compared with that in the IT group (*P* < 0.05).

**Conclusion:**

For osteoporotic IFs (OTA 3.1A2.1–A2.3) in elderly patients, the use of IT aids in decreasing radiographic complications, but the between-group functional outcomes showed no significant difference.

## Background

With the aging population, the incidence of intertrochanteric fractures (IFs) is increasing [1–3]. Because elderly patients have comorbidities, the mortality rate from IFs in these patients is 12 to 41% within 6 months [4, 5]. Studies have shown that early surgical intervention (within 24 h) can significantly reduce mortality [5]. Intramedullary fixation, with the advantages of withstanding the stress of the axis shift, good anti-fatigue performance, smaller incisions, and less damage to the local blood supply, is the most current treatment for IFs [2, 6].

Most elderly patients following IFs, without osteoporosis, are treated surgically (IT or PFNA) and have good surgical results [7, 8]. Nevertheless, some elderly patients with osteoporotic IFs undergo IT or PFNA processes and are most likely to have poor surgical results [9, 10]. Data from recent studies have confirmed that an increasing number of elderly patients have been presenting with osteoporotic IFs [11–16]. In China, for example, the number of fractures attributable to osteoporosis will increase between 2017 and 2040 by approximate 129% if the fracture rate (5.6%) remains constant and is estimated to be 45.0 million. The prevalence of osteoporotic fractures in Europe in 2017 was estimated at 25.0 million. It may, therefore, be very meaningful to know what happens to this group of patients.

The aim of this study was to compare the clinical and radiographic outcomes of InterTAN nail (IT) and proximal femoral nail anti-rotation (PFNA) in the management of IFs (OTA 3.1A2.1–A2.3) in elderly patients with osteoporosis with a 3-year minimum follow-up.

## Methods

### Study population

This study is a retrospective review from a trauma database (Jinshan Hospital, Fudan University). The variability tested of descriptive characteristics of patients in Table 2 was statistically non-significant between the groups (*P* > 0.05). Patient age was divided into five categories: 65–69, 70–79, 80–89, 90 years, or older. Chronic illness burdens before IFs were similar for the two groups as per comparison of the number of comorbidities. Osteoporosis was defined as a bone mineral density (BMD) T-score value ≤ − 2.5 at the contralateral femoral neck.

### Inclusion and exclusion criteria

The inclusion criteria were as follows: freshly closed IFs (OTA 3.1A2.1–A2.3) following a fall, age ranging from 65 to 92 years, a BMD T-score value ≤ − 2.5 at the contralateral femoral neck, and IT or PFNA fixation (IT, diameter: lag screw, 11 mm; compression screw, 7 mm; composite screw, 15.5 mm; length, normal; number of proximal/distal screws, 2/1, Smith & Nephew, Memphis, Tennessee; PFNA, proximal diameter, 16.5 mm; distal diameter, 9–10 mm; length, 240 or 300 mm; number of proximal/distal screws, 1/1; valgus curvature, 5°, Synthes, Solothurn, Switzerland). Exclusion criteria included multiple fractures, fracture secondary to cancer or major trauma, hip fracture during the previous calendar year, sub-trochanteric fracture or femoral neck fracture, and an American Society of Anesthesiologists (ASA) score of IV or V.

### Surgical methods

All of the patients with osteoporosis received an intravenous injection of cefazolin sodium pentahydrate (2.0 g) 30 min before surgery. The patients were placed on a traction operation bed and received general anesthesia. The limb was neutral. Closed traction reduction was performed under an X-ray machine for all of the patients. The point of needle insertion was slightly medial to the exact tip of the greater trochanter. The tip-apex distance (TAD) was limited to approximately 20 mm. No bone grafting was carried out in any of the cases. In the PFNA group, a small incision was made, and the main nail was rotated into the medullary cavity to stabilize the fracture followed by the locator, locking the nail proximally or distally. In the IT group, the installation of compressing intramedullary interlocking nails was performed. The pressurization effect was confirmed. Next, the distal locking screws were installed. All of the surgeries were carried out at our institution by orthopedic surgeons. The technique was identical to that described by Mereddy et al. [17] for PFNA and Ruecker et al. [18] for IT.

### Postoperative management

Passive movement of the quadriceps femoris and ankle joint was performed immediately after surgery. A pneumatic drive pump promoting backflow of the lower limbs was utilized. A once-daily subcutaneous injection of Clexane (4000 AXa IU, Shanghai, China) for the prevention of lower limb venous thrombosis was carried out as early as 6 h postoperatively for seven subsequent days. Twice-daily cefuroxime sodium 1.5 g was used beginning the day before surgery and for three subsequent days. On the third day after surgery, continuous passive motion (CPM; Smith & Nephew, Shanghai, China) was applied to promote the rehabilitation of hip joint function. Postoperative X-rays were used to examine fracture stability. The rehabilitation protocol was identical, and the patients were mobilized on the first postoperative day. Partial weight-bearing as tolerated or restricted weight-bearing was allowed according to the surgeon’s recommendation on the following day.

### Method of assessment

Follow-up occurred 1, 3, 6, and 12 months postoperatively and every year thereafter. The primary endpoint measured was the radiographic outcomes which were obtained at each visit. Major changes of the implant were noted. Electronic medical records and digital radiographs were reviewed by two of us (WY and HZ) to collect all variable outcomes which were assessed by a review (WY). The secondary endpoint was patient-related functional outcomes which were evaluated based on the Harris Hip Score (HHS) by a surgeon-assessor (XCZ). Malunion was defined as less than 50% contact between the proximal and distal fragments or collodiaphyseal angle (CCD) of less than 120°. Bone union was defined by the following radiographic parameters: restoration of cortical continuity, loss of a clear fracture line, and presence of callus. Non-union which means non-union of the fracture itself, not that of the third fragment, was defined as the state in which disturbed consolidation of a fracture that needs further surgical intervention or a prolonged healing time of more than 12 months or more. TAD was measured based on the description of Li et al. [19]. A literature review was then conducted to identify similar studies and to compare the functional and radiographic outcomes of two devices.

### Statistical analysis

All statistical analysis was performed using SPSS (SPSS statistical package; version 22.0.0). Case characteristics were summarized using descriptive statistics, including the mean (SD), or median (minimum-maximum) for continuous variables and the frequency (percent) for categorical variables. Independent samples *t*-test and chi-square test were used to measure the differences in bi-variate analyses. For all comparisons, statistical significance was assigned at *P* < 0.05.

## Results

### General data comparison

**Fig. 1:**
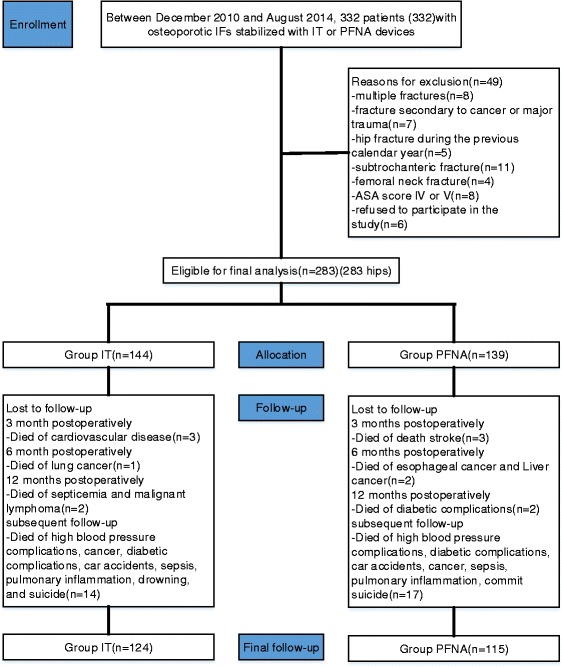
Flow diagram demonstrating methods for identification of studies to assess elderly osteoporosis patients with IFs (OTA 3.1A2.1–A2.3) fixed by an InterTAN nail (IT) or a proximal femoral nail anti-rotation (PFNA) device

**Fig. 2:**
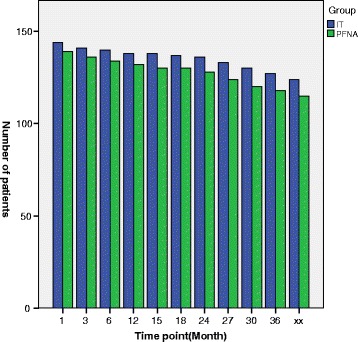
The bar chart clearly shows the changes of the number of patients being lost to follow-up at each time point. The “xx” on the bar chart represents “final follow-up”

From December 2010 to August 2014, 283 patients (283 hips) with osteoporotic IFs (OTA 3.1A2.1–A2.3) stabilized with IT or PFNA devices were enrolled in this study (Figs. 1 and 2, Table 1). The average age was 76.1 years (Table 2). There was no perioperative mortality. During the next follow-up period, there were 41 deaths recorded. No between-group significant differences were noted in terms of the average amount of bleeding, average operation time, and average length of stay. Perioperative medical complications occurred in 20 cases: 7 cases of recurrence of heart disease, 3 cases of pulmonary infection, 2 cases of urinary retention, 4 cases of cerebral infarction, and 2 cases of gastrointestinal dysfunction. The symptoms improved after consultation with relevant departments. The mean follow-up period was 38.8 months (range, 36–43 months) (Tables 3).

**Table 1:** The number of patients being lost to follow-up at each time point

	1	3	6	12	15	18	24	27	30	36	F
IT	144	141	140	138	138	137	136	133	130	127	124
PFNA	139	136	134	132	130	130	128	124	120	118	115
N	0	6	3	4	2	1	3	7	7	5	6

*N* the number of patients being lost to follow-up, *F* final follow-up

**Table 2:** Patient demographics and outcomes

Variable	IT^a^ (*n* = 144)	PFNA^b^ (*n* = 139)	*P* value
Sex, no. M/F	64/80	53/86	0.281*^c^
Age (years)			0.839*^d^
65–69	40	36	
70–79	76	74	
80–89	22	20	
90–92	6	9	
BMI, kg/m^2^	26.1 ± 2.1	25.7 ± 1.9	0.130*^e^
BMD	−3.7 ± 0.3	−3.7 ± 0.4	0.179*^e^
Medical complications	9	7	0.658*^c^
Side, no. Left/right	81/63	72/67	0.453*^c^
OTA fracture type, no.			0.679*^d^
31A2.1	44	36	
31A2.2	72	75	
31A2.3	28	28	
ASA classification			0.501*^d^
1	23	25	
2	65	57	
3	49	54	
4	7	3	
Injury operation interval			0.702*^d^
< 24 h	22	23	
24–48 h	77	79	
48–72 h	29	27	
> 72 h	16	10	
Follow-up (months)	38.7 ± 3.55	39.1 ± 3.43	0.408*^e^

*IT* InterTAN nail, *PFNA* proximal femoral nail anti-rotation, *ASA* American Society of Anesthesiologists, *BMI* body mass index, *BMD* bone mineral density

*No statistically significant values

^a^Smith & Nephew, Memphis, Tennessee

^b^Synthes, Solothurn, Switzerland

^c^Analyzed using chi-square test

^d^Analyzed using the Mann-Whitney test

^e^Analyzed using independent samples *t*-test

**Table 3:** Operation variables

Variable	IT^a^ (*n* = 144)	PFNA^b^ (*n* = 139)	*P* value
Reduction results, no.			0.552*^c^
Anatomical	82	84	
Acceptable	62	55	
Poor	0	0	
Implant position, no.			0.948*^c^
Optimal	101	97	
Suboptimal	43	42	
TAD, mm	19.9 ± 1.25	20.2 ± 1.36	0.144*^d^
Length of stay (days)	11.9 ± 1.62	11.67 ± 1.88	0.21*^d^
Operation time (min)	67.2 ± 3.98	68.9 ± 4.27	0.848*^d^
Blood loss (ml)	180.7 ± 23.03	185.1 ± 18.96	0.078*^d^

*TAD* tip-apex distance, *IT* InterTAN nail, *PFNA* proximal femoral nail anti-rotation

*No statistically significant values

^a^Smith & Nephew, Memphis, Tennessee

^b^Synthes, Solothurn, Switzerland

^c^Analyzed using the Mann-Whitney test

^d^Analyzed using independent samples *t*-test

### Clinical outcome

HHS was used to evaluate the functional outcome. In the IT group, the mean HHS at the last follow-up was 72.2 ± 7.27. In the PFNA group, the mean HHS was 72.4 ± 7.20 at the last follow-up. With regard to the thigh pain in the distribution of the lateral cutaneous nerve of the thigh following surgery in the two cohorts, there was a lower complication rate in IT-treated patients compared with PFNA-treated patients (3.5 vs. 9.4%, *P* = 0.043). Although there was notable difference in thigh pain between groups, no between-group significant difference in HHS was observed at each follow-up.

### Radiographic outcome

Between-group significant differences were noted in fixation failure (cut-out, femoral shaft fracture distal or around the tip of the main nail, varus collapse of the femoral head), which is showed in Table 4.

**Table 4:** Long-term follow-up outcomes

Variable	IT^a^ (*n* = 144)	PFNA^b^ (*n* = 139)	*P* value
Screw cut-out	3	11	0.024*^c^
Femoral shaft fracture^#^	3	12	0.014*^c^
Delayed union	4	5	0.709^c^
Malunion	2	0	0.498^d^
Non-union	2	0	0.498^d^
Varus collapse of the femoral head	1	8	0.020*^c^
Lower limb shortening(> 1.5 cm)	2	2	0.972^c^
Heterotopic ossification	3	1	0.326^c^
Avascular necrosis of the femoral head	1	1	1.000^d^
Postoperative thigh pain	5	13	0.043^*c^
Lower extremity venous thrombosis	7	6	0.797^c^
HHS at the last follow-up	72.2 ± 7.27	72.4 ± 7.20	0.842^e^
Mortality	19	22	0.529^c^

*IT* InterTAN nail, *PFNA* proximal femoral nail anti-rotation, *HHS* Harris Hip Score

*Statistically significant values

^#^Displaced shaft fractures distal or around the tip of the main nail

^a^Smith & Nephew, Memphis, Tennessee

^b^Synthes, Solothurn, Switzerland

^c^Analyzed using the Pearson chi-squared test

^d^Analyzed using Fisher’s exact test

^e^Analyzed using independent samples *t*-test

The incidence of fixation failures in IT-treated patients was significantly lower than PFNA-treated patients (cut-out 2.1 vs. 7.9%, *P* = 0.024; femoral shaft fracture distal or around the tip of the main nail, 2.1 vs. 8.6%, *P* = 0.014; varus collapse of the femoral head, 0.7 vs. 5.8%, *P* = 0.020, respectively). The average TAD was similar between the IT-treated and PFNA-treated patients (19.9 mm [range, 17–25 mm] vs. 20.2 mm [range, 16–23 mm], *P* = 0.144). Among IT-treated patients, fixation failed in 7 fractures (4.8%), which consisted of 3 cut-outs, 1 varus collapse of the femoral head requiring further surgery intervention as of the 2-year follow-up, 1 femoral shaft fracture distal to the tip of the main nail, and 2 femoral shaft fractures around the tip of the main nail requiring the revision surgeries 1.5 years after surgery. Three patients with failed internal fixation received surgical intervention (arthroplasty) secondary to varus collapse with a screw cut-out. Three patients developed varus collapse and failed to have further surgery. One patient with varus collapse without screw cut-out refused further surgical intervention due to the lack of economic ability. Among PFNA-treated patients, fixation failed in 31 fractures (22.3%), which consisted of 11 cut-outs, 8 varus collapses of the femoral head requiring further surgical intervention as of the 3-year follow-up, and 4 femoral shaft fractures distal to the tip of the main nail and 8 femoral shaft fractures around the tip of the main nail requiring revision surgeries 2 years after surgery. There was significant difference with respect to the mean time to failure between IT-treated and PFNA-treated cohorts (18 months [range, 8–19 months] vs. 8 months [range, 2–9 months], *P* = 0.000).

## Discussion

The most important finding of the current research was that the use of IT aids in decreasing radiographic complications, but the between-group functional outcomes showed no significant difference.

Increasingly, PFNA devices are being used to manage IFs [19]. Compared with IT devices, most previous studies have shown a higher radiographic complication rate using PFNA devices [3, 20]. However, in those studies, there was no significant difference in the rate of varus collapse between the PFNA and IT devices. In a series of 225 cases with IFs fixed with a PFNA or IT device, investigators described that varus collapse occurred in 4.9 or 5.5% of cases, respectively (*P* = 0.95) [14]. Inconsistent with previous reports, in this study, the PFNA was related to radiographic complications such as varus collapse (5.8%), femoral shaft fracture distal or around the tip of the main nail (8.6%), and screw cut-out (7.9%). Our findings are comparable to the results published in other studies [15, 21–23]. Nevertheless, the management of IFs remains controversial, particularly in utilizing PFNA or IT devices [3].

PFNA has 6° angles of the valgus, which is consistent with the proximal femoral anatomy. The proximal end of the main nail of IT adopts the trapezoidal cross section design and has a 4° angle of the valgus. This type of design made it easy to insert the main nail during the operation [14]. The PFNA spiral blade provided the greatest degree surrounding the bone filling, which prevented the rotation of the fracture and implant cut-out [15]. IT with a unique combination of interlocking nails could generate a maximum of 15 mm of non-rotating axial compression. The design of the long tip and groove of the main nail of PFNA made its insertion more convenient and avoided the local concentration of stress [16]. IT, with the distal bifurcation design of the main nail, dispersed the stress of the femoral anterior cortex, avoided periprosthetic fractures, and reduced the incidence of postoperative thigh pain [14, 21].

In addition, because of the intact femur anterior arch of elderly patients with osteoporotic IFs and the invariant intramedullary nail shape, if the main nail of the PFNA must be forced into the medullary cavity, it may cause the nail tip break through to the femoral anterior cortex or result in the risk of fracture [22, 23]. We encountered difficulties with the insertion of the main nail, which was considered to have a significant relationship with the angle of the anterior arch of the femur. Postoperative thigh pain was observed between groups. The possible explanation was that the distal intramedullary nail contacted the femur anterior cortex to generate friction, resulting in stress concentration, but no iatrogenic fracture was noted in the recent follow-ups [22, 24]. de Landevoisin et al. [25] reported 102 PFNA-treated cases and discovered one with acetabular penetration, 2 nail-related fractures, and 5 blade back-outs (2 mm) responsible for pain, but there was no significant effect on post-reduction maintenance and fracture healing.

Although TAD is the strongest predictor of the cut-out of a lag screw, increased age and osteoporosis might also be considered strong predictors. Some previously published studies underlined TAD as a predictor of implant failure [16, 21–23]. However, these studies failed to explore the relationship of age and osteoporosis to each implant category [16, 26].

Although intramedullary implants are better than conventional extramedullary implants, still they carry the variable rate of complications in form of femoral shaft fractures distal or around the tip of the main nail, cut-out, more revision surgeries, and finally decreased functional outcome [21, 25]. In the current study, 12 (8.6%) observed for PFNA had femoral shaft fractures distal or around the tip of the main nail. The high shaft fracture rate, despite its theoretically better design, was likely to be attributed to this fact that the looseness of distal locking screws existed, which was confirmed by the second revision surgery and would result in stress concentration at the tip of the main nail. The underlying cause may be osteoporosis, which results in the looseness of the PFNA fixation. In contrast, this high femoral shaft fracture rate is less observed in IT devices. Also, 11 cut-outs were observed in the PFNA-treated patients with osteoporosis. In patients with early postoperative excessive activity related to cut-outs, we removed the PFNA device and implemented traction treatment. Based on the outcome of comparative analysis, no statistically significant differences existed between groups in terms of fracture healing or HHS. However, the IT device was superior to the PFNA device in these aspects (cut-out, femoral shaft fracture distal or around the tip of the main nail, varus collapse of the femoral head, and postoperative thigh pain), which could be considered an important factor when deciding the superiority of any particular implant.

Caution needs to be taken when interpreting relatively high failure rates of internal fixation in the current study, as several factors have been identified. Firstly, stable fracture patterns in osteoporotic bone in conjunction with the quality of the reduction and screw positioning in the femoral head-neck fragment did not predispose to implant cut-out, femoral shaft fracture, or varus collapse of the femoral head. Often the failure is due to inadequate reduction, poorly placed femoral head-neck screw, or mal-positioning of the implant. Of the 38 cases of failure in our series, three fractures were poorly reduced, six femoral head-neck screws were poorly placed, and three main nails were inclined to the lateral wall. Secondly, early functional exercise and high activity levels might increase the risk of implant failure. Appropriate delayed partial weight-bearing might have a strong positive effect on the outcome. Thirdly, patient ages and time to surgery played an important role in fragility fracture treatment. We noted implant failure tended to occur in patients aged 80 years or older or patients with surgical intervention time more than 72 h in our study. Fourthly, TAD less than 19 mm might be associated with failure rates. The finding might provide a guide for surgeons in optimal screw position for reducing the risk of mechanical failure when performing reduction and fixation of IFs.

Our study is limited by its retrospective nature. Furthermore, patient- and surgeon-related confounders may have existed. Nevertheless, both groups were well matched, which allowed us to draw the conclusion that the between-implant differences observed were not related to the patients’ demographics or severity of fracture.

## Conclusions

There is a compelling evidence that IT might be more appropriate in stabilizing osteoporotic IFs (OTA 3.1A2.1–A2.3). Although we have shown a higher complication rate than in some previous series, the application of IT for the fixation of osteoporotic IFs (OTA 3.1A2.1–A2.3) might be the preferred choice compared with PFNA. However, the undeniable fact is that the current study was unable to reveal whether the IT or the PFNA is the optimal treatment for osteoporotic IFs (OTA 3.1A2.1–A2.3). As such, the authors recommend that further prospective randomized trials would be mandatory.

## Abbreviations

ASA: American Society of Anesthesiologists
BMD: Bone mineral density
CPM: Continuous passive motion
HHS: Harris Hip Score
IFs: Intertrochanteric fractures
IT: InterTAN nail
PFNA: Proximal femoral nail anti-rotation
SD: Standard deviation
TAD: Tip-apex distance
